# Additive Effects of L-Ornithine on Preferences to Basic Taste Solutions in Mice

**DOI:** 10.3390/nu13113749

**Published:** 2021-10-23

**Authors:** Haruno Mizuta, Natsuko Kumamoto, Shinya Ugawa, Takashi Yamamoto

**Affiliations:** 1 Department of Nutrition, Faculty of Health Sciences, Kio University, 4-2-4 Umami-naka, Koryo, Kitakatsuragi, Nara 635-0832, Japan; h.mizuta@kio.ac.jp; 2 Department of Anatomy and Neuroscience, Graduate School of Medical Sciences, Nagoya City University, 1 Kawasumi, Mizuho-cho, Mizuho-ku, Nagoya 467-8601, Japan; natsuko@med.nagoya-cu.ac.jp (N.K.); ugawa@med.nagoya-cu.ac.jp (S.U.); 3 Health Science Research Center, Kio University, 4-2-4 Umami-naka, Koryo, Kitakatsuragi, Nara 635-0832, Japan

**Keywords:** L-ornithine, *kokumi*, basic taste solutions, taste preference, electrophysiology, chorda tympani, immunohistochemistry, GPRC6A

## Abstract

In addition to the taste receptors corresponding to the six basic taste qualities—sweet, salty, sour, bitter, umami, and fatty—another type of taste receptor, calcium-sensing receptor (CaSR), is found in taste-bud cells. CaSR is called the ‘*kokumi*’ receptor because its agonists increase sweet, salty and umami tastes to induce ‘*koku*’, a Japanese word meaning the enhancement of flavor characters such as thickness, mouthfulness, and continuity. *Koku* is an important factor for enhancing food palatability. However, it is not well known whether other *kokumi*-receptors and substances exist. Here, we show that ornithine (L-ornithine but not D-ornithine) at low concentrations that do not elicit a taste of its own, enhances preferences to sweet, salty, umami, and fat taste solutions in mice. Increased preference to monosodium glutamate (MSG) was the most dominant effect. Antagonists of G-protein-coupled receptor family C group 6 subtype A (GPRC6A) abolished the additive effect of ornithine on MSG solutions. The additive effects of ornithine on taste stimuli are thought to occur in the oral cavity, and are not considered post-oral events because ornithine’s effects were confirmed in a brief-exposure test. Moreover, the additive effects of ornithine and the action of the antagonist were verified in electrophysiological taste nerve responses. Immunohistochemical analysis implied that GPRC6A was expressed in subsets of type II and type III taste cells of mouse circumvallate papillae. These results are in good agreement with those reported for taste modulation involving CaSR and its agonists. The present study suggests that ornithine is a *kokumi* substance and GPRC6A is a newly identified *kokumi* receptor.

## 1. Introduction

Each food culture in the world contains traditional wisdom that local people have accumulated over their history using foodstuffs specific to their geographical and climatic conditions. For example, Japanese foods are traditionally cooked with *‘dashi*’ (a Japanese boiled extract or soup stock) as a seasoning to increase appetitive flavors. *Dashi* has been used in Japanese cuisine for more than 1000 years, suggesting a base of traditional Japanese food [1,2,3]. Examples of *dashi* come from ‘*kombu*’ (or ‘*konbu*’, dried kelp seaweed), ‘*katsuobushi*’ (dried bonito flakes) and dried ‘*shiitake*’ mushroom. *Dashi* has a very simple and subtle taste compared with the complex taste of soup stock in China and Western countries [2]. In 1909, Ikeda reported that the key element of *kombu-dashi* is glutamic acid [4], which was modified to monosodium glutamate (MSG) by replacing the H ions with Na ions [4]. This finding was followed by the discovery of inosine monophosphate (IMP) from *katsuo-dashi* [5] and guanylate monophosphate (GMP) from *shiitake-dashi* [6]. These three substances induce a peculiar taste called umami in sensory evaluations and make food delicious, and are now collectively referred to as umami substances.

Partly because the flavor of Japanese *dashi* is subtle and mild, Japanese people discovered a technique for making dashi stronger and more delicious by mixing *kombu-dashi* and *katsuo-dashi*, a process that exploits the scientific principle of synergy in combining the umami of MSG and IMP [6,7,8,9]. Japanese people may describe the mixed *dashi* as having ‘*koku*’ in comparison to simple *kombu*- or *katsuo*-dashi. Furthermore, adding soy sauce or *miso* paste (fermented beans), both rich in various amino acids [2], to the mixed *dashi* will boost the expression of *koku*. *Koku* is a Japanese word literally derived from ‘strong’, ‘rich’ or ‘concentrated’. An essential condition for inducing *koku* is the existence of glutamate and other varieties of ions and molecules, including foodstuffs rich in free amino acids. The concept of *koku* applies to edibles with extensive flavor characteristics induced by complex interactions among different sensory modalities that in many cases lead to a state of strong palatability [10]. Therefore, *koku* is not one of the sensory modalities, such as taste, smell, touch, and pain, nor is it a qualitative aspect of sensations, such as sweet, sour, salty, bitter, warm, cold, hot, and astringent. *Koku* is related to the quantitative aspect of sensations and positive hedonics. To scientifically evaluate *koku* in human sensory tests, attributes of *koku* are assessed, such as thickness (strength, concentration, richness, complexity, depth), continuity (lingeringness, aftertaste), mouthfulness (the reinforcement of a taste sensation throughout the mouth with or without a tongue-coating sensation), mildness (smoothness, balance, harmony), and punch (impact) [10,11,12,13,14].

*Koku* can be induced by interactions among tastes without the participation of other sensory modalities. This phenomenon was characterized in a pioneering study performed by Ueda et al. in 1990. According to their paper [12], “addition of a small amount of extract of garlic to umami solution (MSG + IMP) enhances its flavor characters, such as thickness, mouthfulness, and continuity”. These characteristics are the same as those describing *koku*; however, Ueda et al. coined a new term ‘*kokumi*’ to denote *koku* that is induced only by modification of taste information by adding substances, even at low concentrations, without eliciting a taste of their own [13]. It is noted here that *kokumi* is neither a unique taste nor a different kind of *koku*. Such substances are called *kokumi* substances, synonymous to ‘*koku*-inducing substances based on taste interactions’. If one or more specific *kokumi* substance co-exists in foods containing umami substances, the food becomes very delicious with *koku*.

Concerning the identification of *kokumi* substances, garlic extract was first studied [12], and then glutathione was investigated by Ueda et al. [15] in humans and by Yamamoto et al. [16] in mice. Glutathione is a tripeptide consisting of glutamic acid, cysteine and glycine (γ-glutamyl-cysteinyl-glycine, γ-Glu-Cys-Gly). In a human sensory test, Ueda et al. [15] evaluated a simple sample consisting of an umami solution containing 0.05% each of MSG and IMP, and reported that this peptide increased the flavor characteristics of the umami solution. They reported that the increased flavor (or enhanced deliciousness or savoriness) of the umami solution could be expressed by such terms as thickness, continuity, and mouthfulness.

The calcium-sensing receptor (CaSR) is a class C G-protein-coupled receptor that is expressed in mammalian taste-bud cells [17,18,19]. Ohsu et al. [13] found that γ-glutamyl-valyl-glycine (γ-Glu-Val-Gly) was the most active CaSR agonist among 46 γ-glutamyl peptides, including glutathione. Moreover, the most prominent *koku* was observed in a human sensory test when γ-Glu-Val-Gly was mixed independently with sucrose, NaCl, or MSG, and commercially available chicken consommé. Experiments in mice also showed that these *kokumi* substances induce responses in CaSR-expressing taste cells [18]. Interestingly, even sub-threshold concentrations of these substances are effective, indicating that the taste qualities of these substances are not required [13]. The CaSR expressed in taste-bud cells is known as a *kokumi* receptor [13].

With respect to food culture, a common notion among the Japanese is that palatability is markedly enhanced with *koku* when *miso* soup is cooked with corbiculae (*Corbicula fluminea*, *Corbicula japonica*) compared to a simple *miso* soup. It is plausible that a variety of substances including amino acids, succinic acid, and minerals from corbiculae [20] are dissolved in *miso* soup and certain interactions occur with glutamate in the *miso* soup to enhance its palatability. In the present study, we have focused on ornithine because it is plentiful in corbiculae [20], and the taste effectiveness of ornithine has not been previously documented, except for the finding that it decreases bitter taste sensations [21]. Ornithine is a non-essential and non-protein amino acid found broadly in meat, fish, dairy, and eggs. Ornithine plays a central role in the ornithine cycle in the liver and is important for the disposal of excess nitrogen (ammonia). Moreover, ornithine is known to be a potent agonist for the amino acid receptor G-protein-coupled receptor family C group 6 subtype A (GPRC6A) [22,23,24]. Furthermore, the GPRC6A and CaSR proteins are highly homologous [22,23,25]. There are a few reports suggesting the expression of GPRC6A in rodent taste-bud cells [19,26].

Based on the above-mentioned reports, we hypothesize that ornithine is a modulator that induces *koku* after interactions with umami and other taste substances; in other words, ornithine is a *kokumi* substance and GPRC6A is a *kokumi* receptor. Our pilot study involving a human sensory test has confirmed that *miso* soup becomes palatable with *koku* when ornithine is added to the soup [unpublished data]. Although animals may not experience *koku*, we aim to evaluate our hypothesis by performing a detailed behavioral two-bottle preference test and electrophysiological taste nerve recording and an immunohistochemical study on the localization of GPRC6A in taste-bud cells in mice.

## 2. Materials and Methods

### 2.1. Animals

A total of 72 adult male C57BL/6-CrSLC mice, 8 weeks old at the beginning of the experiment, were used. The mice were housed in individual home cages in a temperature-(25 °C) and humidity-(60%) controlled room on a 12:12 h light/dark cycle with lights on at 6:00 a.m. The tests were conducted during the light cycle. Animals had free access to food (CLEA Rodent Diet CE-2, CLEA Japan, Inc., Tokyo, Japan) and tap water, except for the partial restriction during the brief-access tests described below. All animal care and experimental procedures conformed to the guidelines established by the National Institutes of Health, and the experimental protocols were approved by the Institutional Animal Care and Use Committee at Kio University (No. H30-10, 25 February 2019).

### 2.2. Behavioral Experiment: Two-Bottle Preference Tests

A total of 61 mice were used. Each animal was trained to drink distilled water (dw) from a stainless-steel spout connected to a plastic bottle. The preference test was carried out after one week training period. The two-bottle preference test involved simultaneously presenting two bottles to each cage with their spouts separated by 2 cm. The two bottles contained the same taste stimulus (or dw), but either one was mixed with one bottle containing ornithine. (D-ornithine or L-ornithine, Kanto Chemical, Tokyo, Japan). In the long-term test, the positions of the two bottles were switched at 24 h of the 48-h test session to avoid positional preference. In the short-term test, animals were similarly presented with two bottles and allowed to drink for 5 min after overnight water deprivation. After the test, animals were given free access to water until 6:00 p.m., after which the water was removed for the subsequent overnight period. On the next day, the left and right bottles were switched to avoid positional preference and the 5-min test session was repeated. The fluid-filled bottles were weighed before and after testing to measure intake volume. The total intake volume over 48 h or 10 min was divided by 2 to obtain intake volume per day or per 5 min in the long-term and short-term tests, respectively. The degree of preference was expressed as a preference score (=intake of taste solution with ornithine/sum of intake of taste solution with and without ornithine).

We used a variety of taste stimuli, including *miso* soup and six basic taste solutions. *Miso* soup was made by dissolving commercially available *miso* (fermented soybeans) paste (Tokujyou, Takeyamiso Co., Ltd., Tokyo, Japan) at a concentration of 0.7% into hot (90 °C) dw. The *miso* soup was cooled to room temperature and centrifuged (2000× *g*, 5 min). The supernatant was used as a taste stimulus to avoid clogging the spout with precipitates. *Miso* soup with corbiculae was prepared by putting 100 g (about 45 pieces) of commercially available corbiculae into the boiling *miso* soup (300 mL) for 2 min. After cooling to room temperature, the *miso* soup was centrifuged as described above and the supernatant was used as taste stimulus. A mixture of 0.05 M sucrose, 0.05 M NaCl, and 0.05 M MSG was used as a model solution for the *miso* soup. Six basic taste stimuli were used: sucrose (Kanto Chemical), NaCl (Kanto Chemical), citric acid (Kanto Chemical), quinine hydrochloride (QHCl, Kanto Chemical), MSG (Kanto Chemical) and Intralipos (a parenteral stable soybean-oil emulsion, Otsuka Pharmaceutical Factory, Tokyo, Japan). The taste stimuli were dissolved in dw at different concentrations. In addition to MSG, monopotassium glutamic acid (MPG, a gift from Ajinomoto Co., Tokyo, Japan), and inosine monophosphate (IMP, a gift from Ajinomoto Co.) were used as umami substances. L-ornithine was usually added at a concentration of 1 mM, with other concentrations used as required. The Na-channel blocker amiloride (Sigma-Aldrich Co., Tokyo, Japan) was used to reduce the sodium responses of MSG. We presented a range of concentrations of individual tastants to the same group of animals starting from the lowest concentration.

In the experiment using antagonists of GPRC6A, NPS-2143 (Chemscene, Monmouth Junction, NJ, USA), calindol (Cayman Chemical, Ann Arbor, MI, USA), and epigallocatechin gallate (EGCG, Tokyo Chemical Industry, Tokyo, Japan) were used. NPS-2143 and calindol were dissolved in 99.5% ethanol at a concentration of 0.1% and then diluted to each concentration with dw. EGCG was directly prepared in dw.

### 2.3. Taste Nerve Recordings

A total of 5 adult mice were used. The mice were anesthetized by intraperitoneal injection of a combination anesthetic (0.3 mg/kg of medetomidine, 4.0 mg/kg of midazolam, and 5.0 mg/kg of butorphanol). Animals were tracheotomized and secured in a head-holder. The left chorda tympani (CT) nerve was exposed using a lateral approach [27], and was excised as it exited the tympani bulla and dissected away from the underlying tissue. The nerve was then placed onto a platinum wire recording electrode (0.1-mm diameter). An indifferent electrode was placed in contact with nearby exposed tissue. Responses were filtered using a band-pass filter with cutoff frequencies from 40 Hz to 3 kHz and sent to an oscilloscope for visualization. Responses were fed to a digitally controlled summator [28]. The number of discharges was summed over 500-ms epochs with a spike counter (DSE-345; DIA Medical System, Tokyo, Japan) to derive summated responses. The data were stored on a PC and the total spikes over the entire 30-s stimulus period (60 × 500-ms epochs) were counted using the PowerLab system (PowerLab/sp4; AD Instruments, Bella Vista, NSW, Australia) for quantitative analyses. Each stimulus (3 mL) was applied to the anterior dorsal tongue for 30 s followed by a distilled water rinse for at least 60 s. The response to each taste stimulus was expressed relative to the magnitude of responses to 0.1 M NH_4_Cl.

### 2.4. Immunohistochemistry

A total of 11 mice were used to examine which types of taste cell express GPRC6A. Mice were deeply anesthetized with isoflurane and transcardially perfused with saline followed by 2% paraformaldehyde in 0.1 M phosphate buffer (PB). The dissected tongues of mice were soaked in 20% sucrose/phosphate-buffered saline (PBS) overnight at 4 °C, embedded in OCT compound (Sakura Finetechnical, Tokyo, Japan), and then, rapidly frozen. The specimens were sectioned on the coronal plane (20 mm) using a cryostat, mounted on MS-coated glass slides (Matsunami, Osaka, Japan), and air-dried for at least 30 min. Following rinsing in PBS, sections were blocked for 4 h in 5% skim milk, 1% BSA, and 0.2% Triton X-100, and then incubated overnight at 4 °C with an antibody mixture of either rabbit anti-GPRC6A (1:300 dilution; orb385435; Biorbyt, Cambridge, UK) and goat anti-a-gustducin (1:100; LSB4942; LSBio, Seattle, WA, USA), or rabbit anti-GPRC6A (1:300; orb385435; Biorbyt) and goat anti-SNAP-25 (1:100; ab31281; Abcam, Cambridge, UK) diluted in the block solution. After washing in PBS, the sections were incubated with an antibody mixture of Alexa Fluor 594-conjugated anti-rabbit IgG (1:500; A-21207; Thermo Fisher Scientific, Waltham, MA, USA) and Alexa Fluor 488-conjugated anti-goat IgG (1:500; A-11055; Thermo Fisher Scientific) in PBS containing 0.2% Triton X-100 overnight at 4 °C. The sections were then washed in PBS three times, cover-slipped with Fluormount (Diagnostic BioSystems, Pleasanton, CA, USA), and imaged using Nikon A1Rs confocal laser scanning microscope with a 60× Plan Apo 1.40 NA oil immersion objective lens (Nikon, Tokyo, Japan). As for the specificity of the anti-GPRC6A antibody in immunohistochemistry, positive control data are disclosed on the supplier’s website. For negative control, antigen absorption tests with the antigen peptide (orb13066; Biorbyt, Cambridge, UK) were performed according to the standard protocol recommended by the antibody supplier.

### 2.5. Data Analysis

Data are presented as mean ± SE. The Student’s *t*-test (paired, two-tailed) was used to assess statistical differences between two groups. For analyzing more than three groups, we used a one-way ANOVA with Dunnett’s post hoc test, or a repeated measures two-way ANOVA with Tukey’s HSD or Bonferroni post hoc tests for statistical comparisons. *p* values < 0.05 were considered statistically significant except for Bonferroni correction.

## 3. Results

### 3.1. Two-Bottle Preference Test

**Figure 1:**
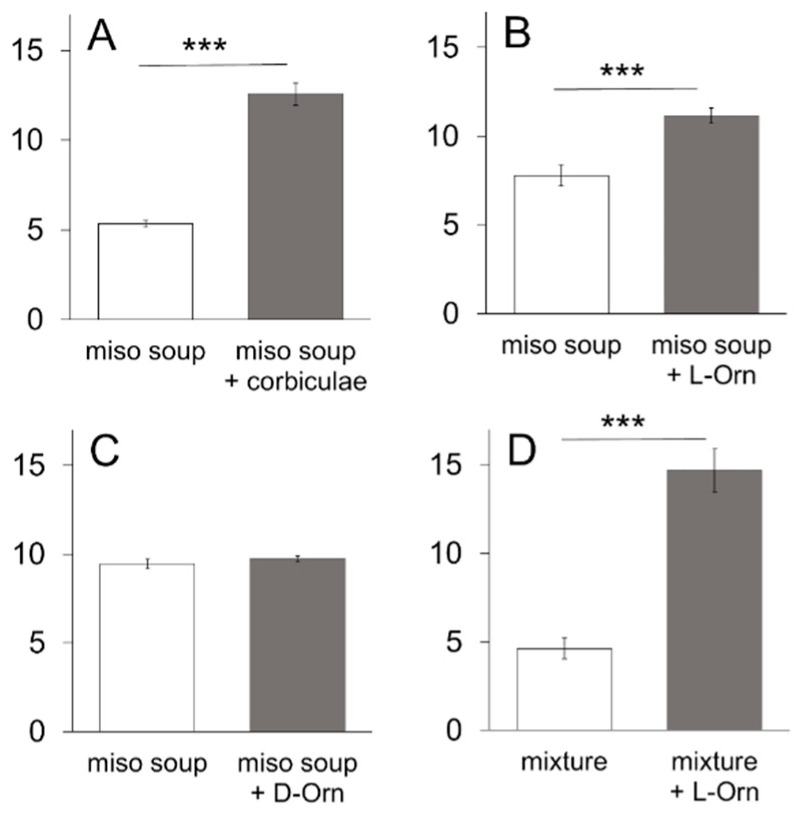
Two-bottle preference test using *miso* soup or a taste mixture in the presence or absence of corbiculae, L-ornithine (L-Orn), or D-ornithine (D-Orn). Fluid intake (mean ± SE; *n* = 8) is compared between *miso* soup and *miso* soup with corbiculae (**A**), L-Orn (**B**), or D-Orn (**C**), and between a taste mixture with or without L-Orn (**D**). The taste mixture is an aqueous solution consisting of 0.05 M MSG, 0.05 M sucrose, and 0.05 M NaCl. *** *p* < 0.001.

When we compared preferences between the supernatant fluid of *miso* soup and that of *miso* soup with corbiculae, the latter soup was significantly (*p* < 0.001, paired *t*-test, two-tailed) preferred to the former (Figure 1A). The supernatant fluid of *miso* soup containing 3 mM L-ornithine was also significantly (*p* < 0.001) preferred to the non-supplemented supernatant fluid (Figure 1B), whereas the supernatant fluid containing 3 mM D-ornithine, an optical isomer of L-ornithine, was not preferred (Figure 1C). We, therefore, used L-ornithine throughout the rest of this study. Since the taste palatability of *miso* soup is thought to arise from a mixture of umami, salty, and sweet tastes, we used an artificial mixture of 0.05 M MSG, 0.05 M NaCl, and 0.05 M sucrose instead of *miso* soup in the next test. As shown in Figure 1D, the mixture containing 3 mM L-ornithine (abbreviated as Orn hereafter) was very significantly (*p* < 0.001) preferred to the non-supplemented mixture.

**Figure 2:**
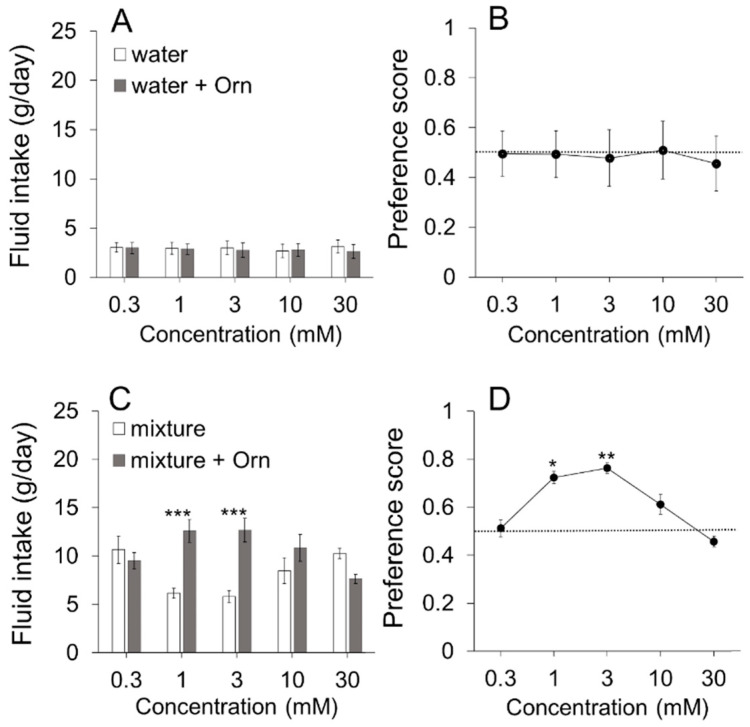
Additive effects of different concentrations of L-ornithine (Orn). (**A**) Water intake with or without five concentrations of Orn. (**B**) Preference score for water with Orn. (**C**) Intake of a mixture of 0.05 M MSG, 0.05 M sucrose, and 0.05 M NaCl with or without Orn. (**D**) Preference scores for the mixture with Orn. Each value is mean ± SE; *n* = 8. * *p* < 0.05, ** *p* < 0.01, *** *p* < 0.001.

To examine whether Orn itself has a palatable taste, we conducted a preference test between water and aqueous solutions containing various Orn concentrations. There was no significant difference between the test liquids at concentrations of Orn ranging from 0.3 to 30 mM (Figure 2A), and therefore no significant difference in preference scores for Orn solutions within this concentration range (Figure 2B). However, although not shown in the figure, 100 mM Orn was rejected (water vs. 100 mM Orn: 4.9 g vs. 0.2 g, *p* < 0.05) with a preference score of 0.20, indicating that higher concentrations of Orn are aversive.

Figure 2C shows the additive effect of different concentrations of Orn on the preference for the artificial mixture described above. Two-way (Solution × Concentration) ANOVA revealed a significant main effect of Solution [F(1,70) = 9.78, *p* < 0.01] and a Solution-Concentration interaction [F(4,70) = 6.4, *p* < 0.001], although there was no main effect of Concentration. Post hoc Tukey’s analysis showed that addition of 1 and 3 mM Orn to the mixture increased intake highly significantly (*p* < 0.001) over the mixture alone. Figure 2D shows preference scores for the mixture with Orn calculated using the same data shown in Figure 2C. One-way ANOVA revealed a significant main effect of Concentration [F(4,28) = 5.81, *p* < 0.01]. Post hoc Dunnett test showed that the mixtures containing 1 and 3 mM Orn were significantly (*p* < 0.05 and *p* < 0.01, respectively) preferred to the mixture containing 0.3 mM Orn taken as control. However, other concentrations of Orn (0.3, 10, and 30 mM) had no effect on preference (Figure 2C,D). In the following experiments, we used Orn at the concentration of 1 mM.

**Figure 3:**
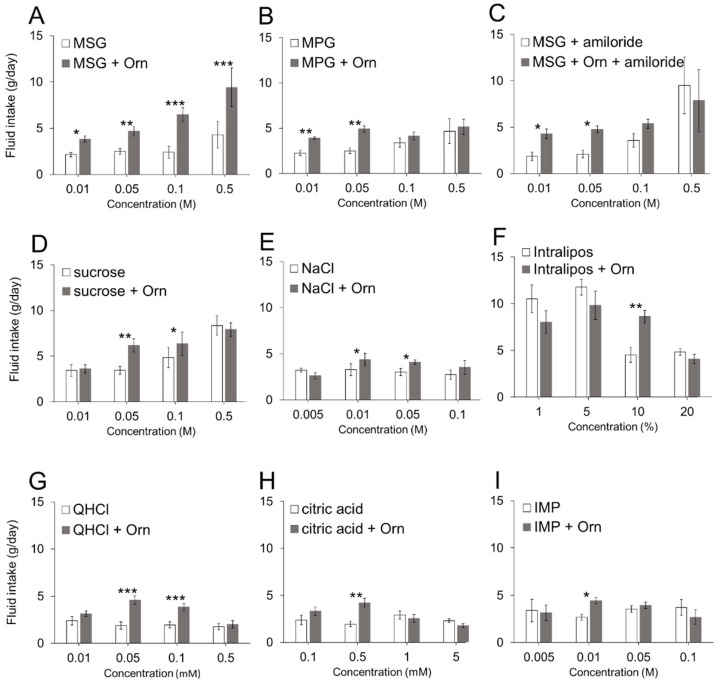
Additive effects of 1 mM L-ornithine (Orn) on different concentrations of taste solutions. Fluid intake (mean ± SE; *n* = 8) with and without Orn is shown for MSG (**A**), MPG (**B**), MSG with 0.01 mM amiloride (**C**), sucrose (**D**), NaCl (**E**), Intralipos (**F**), quinine hydrochloride (QHCl) (**G**), citric acid (**H**), and IMP (**I**). * *p* < 0.05, ** *p* < 0.01, *** *p* < 0.001.

Next, we compared the amount of intake of each taste solution representing the six basic tastes with and without 1 mM Orn (Figure 3). Each graph shows the amount of intake of one of nine taste solutions at four different concentrations with and without 1 mM Orn. Tukey’s multiple comparisons between solutions with and without Orn and four concentrations of solution revealed that Orn increased the intake of each solution at least at one concentration among the four concentrations tested. It is noted here that innately aversive QHCl and citric acid were significantly preferred at certain concentrations with Orn relative to the absence of Orn (Figure 3G,H). Another interesting finding is that the additive effects of Orn differed among umami solutions. The intake of the MSG solution increased at all four concentrations tested (Figure 3A), whereas the intake of the IMP solution increased only at 0.01 M (Figure 3I), and the intake of MPG increased only at 0.01 and 0.05 M (Figure 3B). When amiloride was added to the MSG solution to eliminate the effect of Na ions, an additive effect of Orn was not observed at higher concentrations (0.1 and 0.5 M) (Figure 3C), which was similar to the effect on MPG (Figure 3B).

**Figure 4:**
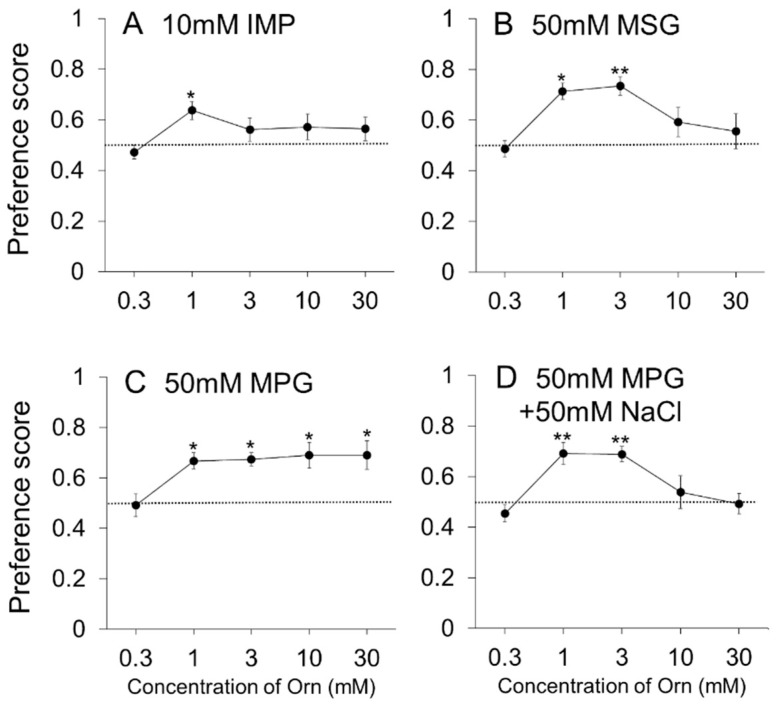
Additive effects of different concentrations of L-ornithine (Orn) on a fixed concentration of umami solutions. Preference scores (mean ± SE; *n* = 8) are shown for 0.01 M IMP with Orn (**A**), 0.05 M MSG with Orn (**B**), 0.05 M MPG with Orn (**C**), and a mixture of 0.05 M MPG and 0.05 M NaCl with Orn (**D**). * *p* < 0.05, ** *p* < 0.01 (vs. preference score when supplemented with 0.3 mM Orn).

Since Orn strongly affected the preference for umami solutions, especially MSG, the next experiment aimed to examine the effects of different concentrations of Orn on fixed concentrations of individual umami compounds. Figure 4A shows that the preference score for 0.01 M IMP increased slightly at 1 mM Orn. One-way ANOVA revealed a significant main effect of Concentration [F(4,28) = 3.21, *p* < 0.05]. Post hoc Dunnett test showed that IMP containing 1 mM Orn was significantly (*p* < 0.05) preferred to IMP containing 0.3 mM Orn taken as control. Figure 4B shows that preference score for 0.05 M MSG increased when 1 and 3 mM Orn was added. One-way ANOVA revealed a significant main effect of Concentration [F(4,28) = 4.11, *p* < 0.01]. Post hoc Dunnett test showed that MSG containing 1 and 3 mM Orn was significantly (*p* < 0.05 and *p* < 0.01, respectively) preferred to MSG containing 0.3 mM Orn. On the other hand, as shown in Figure 4C, Orn increased the preference for 0.05 M MPG at concentrations ranging widely from 1 to 30 mM. One-way ANOVA revealed a significant main effect of Concentration [F(4,28) = 4.15, *p* < 0.01]. Post hoc Dunnett test showed that MPG containing 1, 3, 10 or 30 mM Orn was significantly (*p* < 0.05) preferred to MPG containing 0.3 mM Orn. When 0.05 M NaCl was added to 0.05 M MPG, the effect of 10 and 30 mM Orn on preference was eliminated as shown in Figure 4D, suggesting a disappearance of Orn-induced preference for glutamate in the presence of Na ions at higher concentrations (10 and 30 mM) of Orn. One-way ANOVA revealed a significant main effect of Concentration [F(4,28) = 5.21, *p* < 0.01]. Post hoc Dunnett test showed that 1 or 3 mM Orn increased preference score for the mixture of MSG and NaCl significantly (*p* < 0.01) more than 0.3 mM Orn.

**Figure 5:**
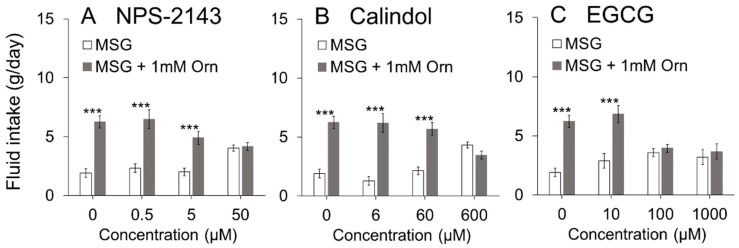
Effects of GPRC6A antagonists on the amount of intake of 0.05 M MSG with and without 1 mM L-ornithine (Orn). Each of the antagonists, (**A**) NPS-2143, (**B**) Calindol and (**C**) EGCG, dose-dependently suppressed. Each of the antagonists (NPS-2143, calindol and EGCG) dose-dependently suppressed Orn-induced preference. Each value is mean ± SE; *n* = 8. *** *p* < 0.001.

It is reported that Orn is a potent agonist of the amino acid receptor, GPRC6A [27,28,29]. Therefore, we examined the effects of GPRC6A antagonists on Orn-induced taste preferences to reveal the possible existence and involvement of GPRC6A in taste-bud cells. Each of the three antagonists (NPS-2143, calindol and EGCG) was added to a 0.05 M MSG solution and the amount of intake of this mixture in the absence and presence of Orn was compared. As shown in Figure 5, all the antagonists dose-dependently suppressed Orn-induced preference. Two-way (Solution × Concentration) ANOVA revealed a significant main effect of Solution [F(1,56) = 66.16, *p* < 0.001] and a Solution-Concentration interaction [F(3,56) = 7.49, *p* < 0.001], although there was no main effect of Concentration. Post hoc Tukey’s analysis showed that the amount of intake of MSG + Orn was much larger (*p*< 0.001) than that of plain MSG when 0.5 and 5 µM NPS-2143 was added; however, addition of 50 µM abolished the Orn-induced MSG preference. Essentially the same effect was observed for calindol (Figure 5B). Two-way ANOVA revealed a significant main effect of Solution [F(1,56) = 63.58, *p* < 0.001] and a Solution-Concentration interaction [F(3,56) = 12.2, *p* < 0.001], although there was no main effect of Concentration. Post hoc analysis showed that there was no difference in the amount of intake between MSG + Orn and plain MSG only when 600 µM calindol was added. Figure 5C shows the effects of EGCG. Two-way ANOVA revealed a significant main effect of Solution [F(1,56) = 31.05, *p* < 0.001] and a Solution-Concentration interaction [F(3,56) = 6.89, *p* < 0.001], although there was no main effect of Concentration. Post hoc analysis showed that there was no difference in the amount of intake between MSG + Orn and MSG when 100 and 1000 µM EGCG was added.

**Figure 6:**
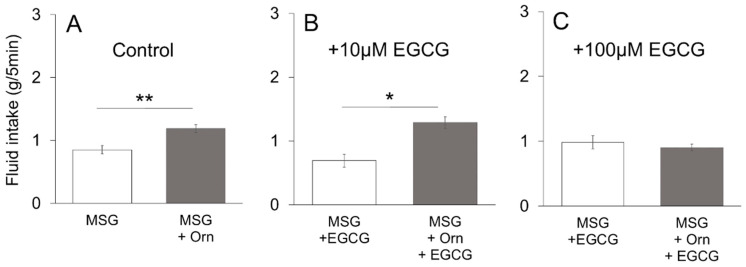
Brief-exposure (5 min) two-bottle preference test for 0.05 M MSG with and without 1 mM L-ornithine (Orn) and effects of EGCG. Intake of MSG increased by addition of Orn in the absence of the GPRC6A antagonist (**A**), or in the presence of 10 µM EGCG (**B**), 100 µM EGCG (**C**); however, the effect of Orn was blocked in the presence of 100 µM EGCG. Each value is mean ± SE; *n* = 8. * *p* < 0.05, ** *p* < 0.01.

The two-bottle brief (5 min) exposure preference test was conducted to determine if the increased preference by addition of Orn was due to an intra-oral event rather than post-oral consequences. As shown in Figure 6A, the intake amount of 0.05 M MSG with Orn was significantly (*p* < 0.01, paired *t*-test, two-tailed) larger than in the absence of Orn. When the GPRC6A antagonist EGCG was added to MSG solutions with and without Orn, Orn significantly (*p* < 0.05) increased the amount of intake in the presence of 10 µM EGCG (Figure 6B), whereas Orn did not produce a significant change in intake in the presence of 100 µM EGCG (Figure 6C). These results are very consistent with those shown in the long-term preference test shown in Figure 5C.

### 3.2. Taste Nerve Recording

**Figure 7:**
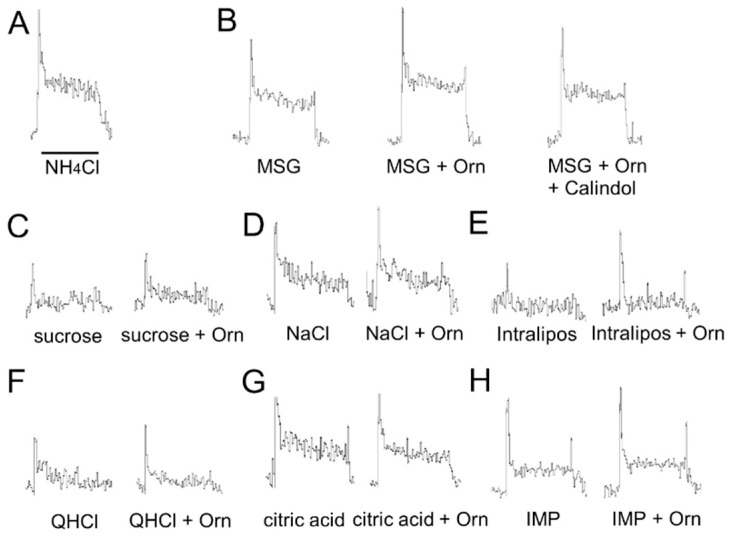
Sample recordings of chorda tympani responses to taste stimuli with and without 1 mM L-ornithine (Orn). (**A**) Responses to 0.1 M NH_4_Cl, (**B**) Responses to 0.05 M MSG, MSG + Orn, and MSG + Orn + 0.6 mM calindol. (**C**) Responses to 0.05 M sucrose with and without Orn. (**D**) 0.05M NaCl with and without Orn. (**E**) Responses to 10% Intralipos with and without Orn. (**F**) Responses to 0.02M QHCl with and without Orn. (**G**) Responses to 0.02M citric acid with and without Orn. (H) Responses to 0.01 M IMP with and without Orn. The horizontal bar indicates 30 s.

Sample recordings of chorda tympani responses to taste stimuli with and without Orn are shown in Figure 7. The MSG response increased following the addition of Orn, and almost returned to pre-addition levels after the addition of calindol (Figure 7B). Sucrose, NaCl, Intralipos, and IMP responses appear to be slightly increased in the presence of Orn (Figure 7C–H); however, this small increase was consistently observed among individual responses. Conversely, QHCl and citric acid responses were slightly diminished by addition of Orn (Figure 7F,G).

**Figure 8:**
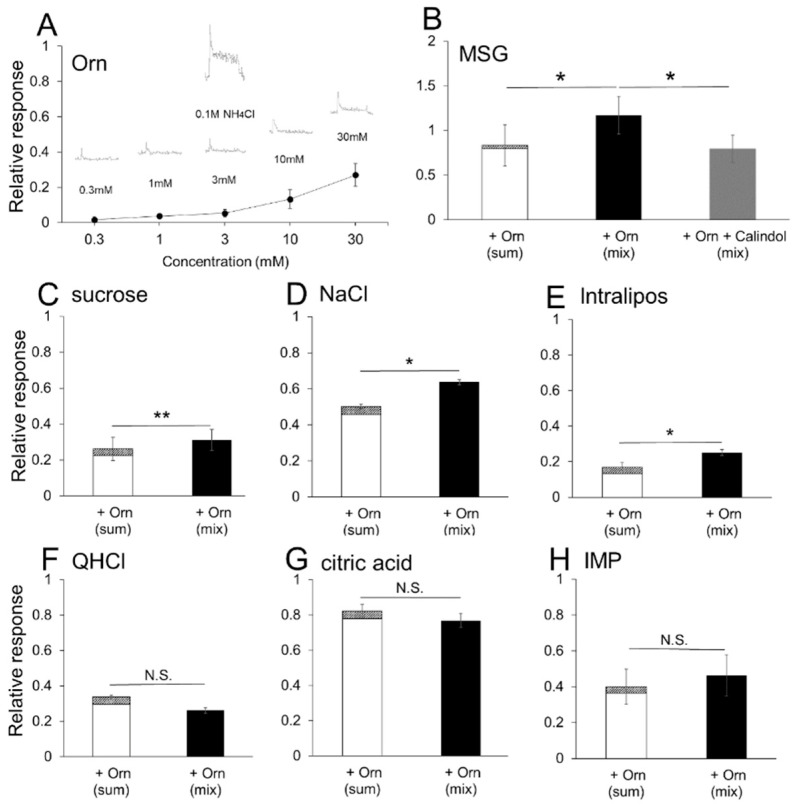
Quantitative representation of the magnitude of chorda tympani responses. (**A**) A concentration-response curve of the effects of aqueous L-ornithine (Orn) solutions on nerve responses. (**B**) The sum of the individual responses to 0.05 M MSG and 1 mM Orn, the response to a binary mixture of 0.05 M MSG and 1 mM Orn, and a mixture of MSG, Orn and 0.6 mM calindol. (**C**) The sum and mixture of 0.05 M sucrose and Orn. (**D**) The sum and mixture of 0.05M NaCl and Orn. (**E**) The sum and mixture of 10% Intralipos and Orn. (**F**) The sum and mixture of 0.02 M QHCl and Orn. (**G**) The sum and mixture of 0.02 M citric acid and Orn. (**H**) The sum and mixture of 0.01 M IMP and Orn. Each value is mean ± SE; *n* = 5. * *p* < 0.017 (after Bonferroni correction) for (**B**), * *p* < 0.05, ** *p* < 0.01 (paired *t*-test, two-tailed) for (**C**–**E**). N.S., not significant.

We compared the mean magnitude of the responses to each stimulus. Orn itself induced small, but dose-dependent increases compared with the standard response to NH_4_Cl (Figure 8A). The responses to Orn at concentrations of 1 and 3 mM, which were used in this study, were negligibly small, corresponding to the result of the present behavioral study indicating that mice could not discriminate between water and Orn at these concentrations (see Figure 2A,B). The responses to binary mixtures of MSG and Orn, sucrose and Orn, NaCl and Orn, and Intralipos and Orn were significantly larger than those to the arithmetic sum of the individual component responses, suggesting the existence of synergism (Figure 8B–E). In the presence of calindol, the response to the mixture of MSG and Orn was equivalent to the sum of MSG and Orn responses (Figure 8B). No significant additive effects of Orn were observed on QHCl, citric acid and IMP responses (Figure 8F–H).

### 3.3. Immunohistochemistry

**Figure 9:**
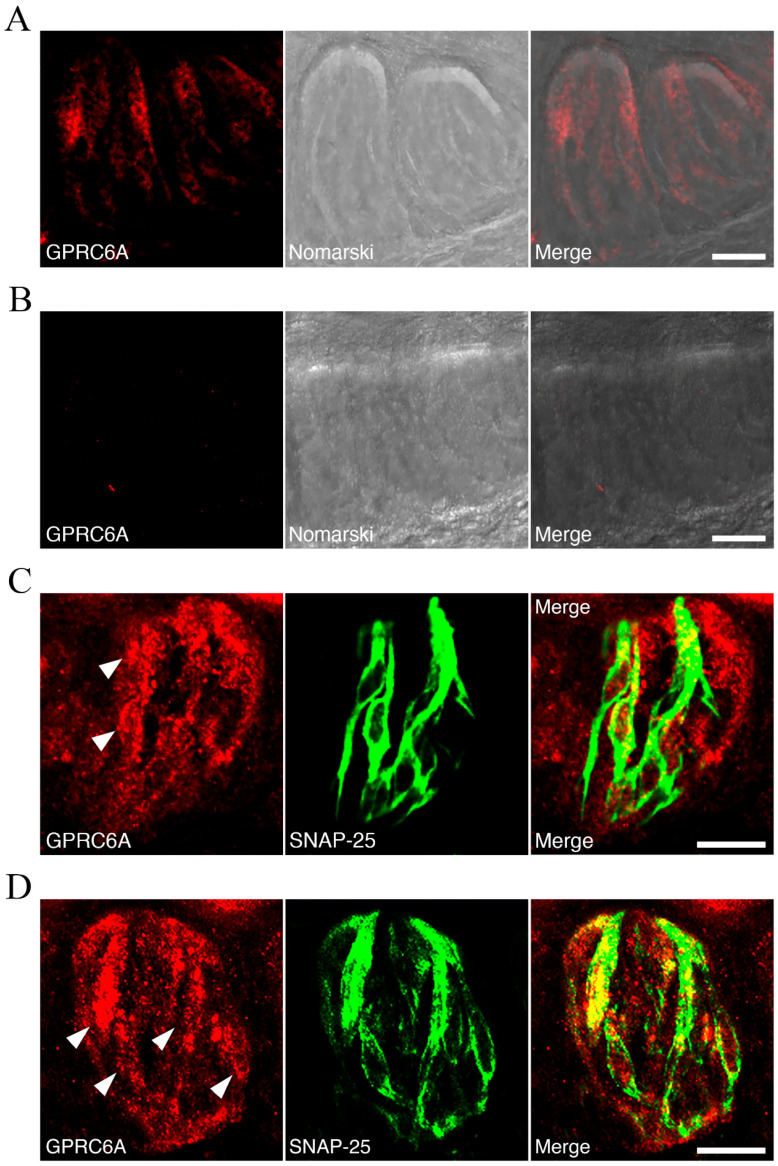
Immunohistochemical analysis of GPRC6A in taste cells of mouse circumvallate papilla taste buds. (**A**,**B**) Fluorescence micrographs of the taste buds immunostained with the anti-GPRC6A antibody in the absence (**A**) or presence (**B**) of the antigen peptide (left panels; middle panels, Nomarski images of the left panels; right panels, merged images of respective right and middle panels). (**C**) Immunoreactivity for GPRC6A is expressed in a small subset of type II taste cells. Shown are immunoreactivity for GPRC6A (red, left panel), immunoreactivity for a-gustducin (green, middle panel) and a merged image of both panels (right panel). Arrowheads indicate GPRC6A-positive type II taste cells. (**D**) Immunoreactivity for GPRC6A is expressed in a subset of type III taste cells. Shown are immunoreactivity for GPRC6A (red, left panel), immunoreactivity for SNAP-25 (green, middle panel) and a merged image of both panels (right). Arrowheads indicate GPRC6A-positive type III taste cells. Scale bars, 20 mm.

To investigate whether GPRC6A was expressed in taste cells of mouse circumvallate papilla taste buds, immunohistochemical study was performed. Moderate to faint immunoreactivity for GPRC6A was detected in a subpopulation of the taste cells (Figure 9A). The immunoreactivity was completely abolished in the presence of the antigen peptide (five times excess blocking peptide to the antibody by weight) (Figure 9B), verifying the specificity of the antibody used. Double-fluorescence immunohistochemistry for GPRC6A and a-gustducin, a maker for type II taste cells [29,30], showed that a small subset of spindle-shaped, a-gustducin-positive taste cells were immunopositive for GPRC6A (Figure 9C). In addition, immunoreactivity for SNAP-25, a maker for type III taste cells [31,32], was detected within the cytoplasm of subgroups of spindle-shaped taste cells, some of which were also GPRC6A-positive (Figure 9D). These data implied that GPRC6A proteins were localized in subpopulations of both type II and type III taste cells in the taste buds.

## 4. Discussion

The present study has demonstrated in mice that low concentrations of Orn that do not produce a taste of their own affected preference behavior when added to each of the six basic taste solutions. Increased preference for MSG was the most dominant effect, which might involve the amino acid receptor GPRC6A, because GPRC6A antagonists abolished the additive effect of Orn on MSG solutions. It is currently known that a similar phenomenon is produced by glutathione and γ-Glu-Val-Gly via CaSR [13,18]. These peptides, together with other substances [33,34,35,36], are called *kokumi* substances [10,12,13] because their addition to umami-rich foods increases the three main attributes of *koku* (thickness, mouthfulness, and continuity) [10,11,12,13]. These substances are agonists of CaSR, which is also known as the *kokumi* receptor [13,18]. The present study is the first to suggest that Orn is an additional *kokumi* substance and that GPRC6A expressed in taste cells is another *kokumi* receptor.

We used the lowest effective concentration of Orn (1 mM) to minimize the possible involvement of the taste of Orn itself throughout the experiments, except when required. However, this concentration may be insufficient to evaluate the overall effects of Orn on taste potentiation. One reason for using a single Orn concentration is to limit the number of combinations of Orn and tastant concentrations. Another reason is that Orn is more effective at lower concentrations (1 and 3 mM) than at higher concentrations (10 and 30 mM) (see Figure 2C,D). However, the occurrence of further potentiation at different concentration combinations remains a possibility.

The main effect of Orn was to enhance the taste of palatable compounds including sucrose, umami substances, and low concentrations of NaCl and Intralipos. As there are no previous reports describing the ability of γ-Glu-Val-Gly to increase the preference for fatty taste, this is the first report describing the novel finding that Orn increases the preference for Intralipos. Orn potentiates only certain concentrations of the tastants. We observed that a single concentration is potentiated while concentrations greater and lower may be unaffected. This finding may be due to the comparison of substances in the absence and presence of Orn. In the case of MSG, if the Orn effect is very strong, MSG in the presence of Orn is preferred to MSG alone over a broad range of concentrations. However, if the Orn effect is moderate, the effect will be dependent on the concentrations of the substances. For example, Orn potentiates sucrose at 0.05 M because high concentrations of sucrose are appealing in the absence of Orn; even if sucrose is slightly potentiated by Orn, no difference in preference is observed. At low sucrose concentrations, such as 0.01 M, the effect of Orn is too small for the animals to differentiate between solutions with and without Orn. Thus, the animals appear to be able to discriminate between the palatability of two stimuli with and without Orn at certain concentrations. If a two-bottle preference test were used with water and a tastant with Orn, we would obtain results that directly reflect the degree of palatability.

The most remarkable effect of 1 mM Orn on preference enhancement was shown for MSG at all the concentrations (0.01 to 0.5 M) tested. However, Orn was not consistently effective with other umami substances: (1) Orn was not very effective in increasing preference for IMP, i.e., among the four concentrations tested, we observed that Orn increased the preference for IMP only at 0.01 M. The reverse was true for glutathione, which is an agonist of the CaSR receptor, where glutathione was found to increase the preference for IMP rather than MSG in mice [16]. (2) Orn increased preference for MPG only at lower concentrations (0.01 and 0.05 M), but was not effective on higher concentrations (0.1 and 0.5 M) of MPG. To examine the possible involvement of sodium ions in the enhancement of preference at 0.1 and 0.5 M glutamate, the Na-channel blocker amiloride was added to both MSG and MSG + Orn solutions. The result showed that preference for 0.1 and 0.5 M MSG was not affected by addition of Orn in the presence of amiloride, indicating that sodium ions play an important role in Orn-induced preference enhancement for 0.1 and 0.5 M glutamate.

Sodium ions are interesting modulators in the sense that they increase the preference for 0.1 and 0.5 M glutamate when a lower concentration (1 mM) of Orn is added, as described above. In contrast, sodium ions decrease the preference for 0.05 M MSG when higher concentrations (10 and 30 mM) of Orn are added (see Figure 4). To confirm the finding that higher concentrations of Orn are not effective, we conducted the same experiment using MPG, which is a glutamate salt containing potassium ions instead of sodium ions, and found that Orn increased the preference for MPG at all the concentrations ranging from 1 to 30 mM. Moreover, the addition of sodium ions (from NaCl) to MPG diminished the increased preference produced by higher concentrations of Orn, suggesting that sodium ions at certain concentrations interfere with the interaction between Orn and glutamate. The role and mechanism of sodium ions in modulating the effects of Orn on glutamate responses will be elucidated in future studies that include data collected from all concentration combinations between Orn and glutamate.

Certain Orn concentrations appear to increase preference for citric acid and QHCl, which are innately aversive [37]. However, it is plausible that citric acid and QHCl responses are decreased by Orn, a possibility that is supported by a human sensory test showing that Orn inhibits bitter tastes [21]. Furthermore, the addition of Orn shortened the quinine retardation-factor in experiments using molecularly imprinted polymers, indicating that Orn directly inhibits the affinity of quinine receptor sites [38].

The present behavioral results were confirmed by chorda tympani nerve responses. Although the difference in magnitude between responses with and without Orn was small when compared to the significant potentiation observed in the preference behavior, this difference was consistently observed among individual responses and statistically significant differences were detected for MSG, sucrose, NaCl, and Intralipos. Taste-induced behavior reflects taste inputs conveyed via all the taste nerves, such as the chorda tympani, glossopharyngeal, greater superficial petrosal, and vagal nerves. In this study, we recorded responses only from the chorda tympani. Although the chorda tympani responses to Intralipos were very small, a significant additive effect of Orn was detected. Intralipos may induce larger responses in the glossopharyngeal nerve innervating the circumvallate and foliate papillae located in the posterior region of the tongue [39]. For other taste stimuli, more dominant ornithine effects may be present in other taste nerves, especially the glossopharyngeal nerve.

The additive effect of Orn on MSG was confirmed in the brief-exposure (5 min) test, in which animals demonstrate preference behavior primarily based on oral sensations, rather than post-ingestive effects. The observed behavioral results were confirmed by taste nerve responses, including (1) increased responses to MSG, IMP, sucrose and Intralipos by addition of Orn; (2) the additive effect of Orn on MSG responses was diminished in the presence of a GPRC6A antagonist, and (3) aqueous Orn solutions elicit very small (if any) effects, which corresponds to the lack of preference behavior for aqueous Orn solutions over a concentration range of 0.3 to 3 mM. These results suggest that almost all the effects induced by Orn are mediated in the periphery, specifically in taste-bud cells and within taste buds.

The CaSR and GPRC6A proteins exhibit a high degree of sequence homology [22,23,24,25], and both are broad-spectrum L-amino acid sensors [40]. However, agonist profiles of these receptors are not identical: basic amino acids (such as lysine, arginine, histidine, and Orn) activate the GPRC6A [25], among which Orn is a potent and specific stimulant of GPRC6A [22,23,24]; whereas aromatic L-amino acids (such as phenylalanine, tryptophan and tyrosine) activate CaSR [40]. To expand on these previous findings, we examined whether GPRC6A plays a crucial role in Orn-induced modulation of taste preference (induction of *kokumi*) using three antagonists of GPRC6A (NPS2143 [41,42], calindol [41,42,43], and EGCG [44]). All three antagonists dose-dependently diminished the additive effect of Orn on the MSG solution. The calcilytic NPS2143 and the calcimimetic calindol have been identified as antagonists of GPRC6A, but are not selective antagonists of GPRC6A as they also act as ligands of CaSR [41]. Therefore, we used the recently reported EGCG, which is contained in green tea, as a selective antagonist for GPRC6A [44]. The chorda tympani recordings showed that the response to MSG was enhanced in the presence of Orn, which was blocked by addition of calindol to the mixture. These behavioral and electrophysiological results suggest that the modulatory effects of Orn are mediated through the activation of GPRC6A.

To understand the underlying mechanism of the modulatory effects of Orn on taste, it is crucially important to understand the expression of GPRC6A in taste-bud cells. Previous studies in this regard are quite limited: (1) Wellendorph et al. [26] reported that GPRC6A mRNA is strongly expressed in the so-called Geschmacksstreifen containing palatal taste buds in rats, (2) Bystrova et al. [19] detected GPRC6A transcripts in individual type I taste cells, but not in type II and type III cells, using the serial multistandard-assisted reverse transcriptase—polymerase chain reaction (SMART-PCR) RNA amplification method in mice. In contrast, immunohistochemical double-labeling studies demonstrated that CaSR is expressed in a subset of type II and type III cells in rats and mice [17], and that CaSR-expressing taste cells represent a subset of cells that are distinct from T1R3-expressing umami or sweet taste receptor cells in mice [18], suggesting that CaSR is not directly involved in umami or sweet taste signaling. Further studies are needed to elucidate the mechanism of *koku* induction by CaSR activation, including the possibility of cell-to-cell signaling within a taste-bud. In the present study, we employed immunohistochemistry to demonstrate that GPRC6A was expressed in both type II and type III taste cells. We are now characterizing the subtypes of cells expressing GPRC6A to determine whether GPRC6A expression is independent of, or co-expressed in, basic taste receptor cells, which will be reported in a future publication. Moreover, further studies using GPRC6A knockout animals, as well as in situ hybridization with GPRC6A sense and antisense probes, will further clarify the role of GPRC6A in taste cells.

No comparative reports are available concerning the additive effects of Orn on basic taste qualities. However, a similar study by Melis et al. [45] investigated the effects of L-arginine (L-Arg) supplementation on the taste perception of five basic taste solutions in humans. Arginine is a precursor to the production of Orn through the urea cycle and the amino acid structure is highly similar to that of Orn. Their study revealed that addition of low concentrations of L-Arg, which did not evoke taste perception in their subjects, enhanced umami taste, NaCl saltiness, and decreased citric acid sourness. Although sweetness of a sucrose solution was also enhanced, about 60% of subjects perceived this solution as bitter rather than sweet. It is known that L-Arg suppresses the bitterness of quinine [38]. These results agree very well with the present findings except for the change in perceived taste quality when L-Arg was added to a sucrose solution.

## 5. Conclusions

In conclusion, the present findings that concentrations of Orn that do not produce a taste of their own increased preference for several umami substances via GPRC6A, are comparable to the previous findings that low concentrations of glutathione and γ-Glu-Val-Gly, which lack a taste effect of their own, increased preference for umami substances via CaSR [13]. Since CaSR is called a *kokumi* receptor and glutathione and γ-Glu-Val-Gly are called *kokumi* substances [13], we propose that GPRC6A is another type of *kokumi* receptor, and that Orn is an additional type of *kokumi* substance. A proper use of a combination of a CaSR agonist (e.g., glutathione) and a GPRC6A agonist (e.g., Orn) would be a fascinating approach to produce very delicious foods with strong *koku*.

## Data Availability

The data that support the findings of this study are available from the corresponding author upon reasonable request.
